# Development, validation, and application of a quantitative volumetric absorptive microsampling–based method in finger prick blood by means of LC-HRMS/MS applicable for adherence monitoring of antipsychotics

**DOI:** 10.1007/s00216-020-03143-0

**Published:** 2021-01-30

**Authors:** Cathy M. Jacobs, Lea Wagmann, Markus R. Meyer

**Affiliations:** Department of Experimental and Clinical Toxicology, Institute of Experimental and Clinical Pharmacology and Toxicology, Center for Molecular Signaling (PZMS), Saarland University, Homburg, Germany

**Keywords:** Adherence monitoring, Microsampling, VAMS, Antipsychotics, LC-HRMS

## Abstract

Volumetric absorptive microsampling (VAMS), an emerging microsampling technique, is expected to overcome some disadvantages of dried blood spots such as volume inaccuracy and influence of hematocrit (HT). This study aimed to develop and evaluate a VAMS-based strategy for quantification of 13 frequently prescribed antipsychotics in finger prick blood within the scope of adherence monitoring to complement already-established qualitative urine analysis. The final workflow consisted of VAMS tip hydration and subsequent precipitation. Samples were analyzed by using reversed-phase ultra-high-performance liquid chromatography and Orbitrap mass spectrometry operated in parallel reaction monitoring mode. The analytical procedure was successfully validated based on international recommendations at three different HT values (20%, 40%, 60%) for most of the analytes. Selectivity and within/between-run accuracy and precision were in accordance with the recommendations in most cases. Internal standard–normalized matrix factor met recommended criteria for all analytes at HT 40%. For the HT values of 20% and 60%, only four substances did not meet the criteria. Dilution integrity was given for all substances, except for olanzapine, allowing a quantification over the whole therapeutic range of selected antipsychotics. Long-term stability in VAMS tips was tested and revealed degradation of five antipsychotic drugs after 1 week of storage at 24 °C. A proof of concept of the applicability of the method was obtained by quantification of a selection of the 13 antipsychotic drugs in VAMS tips and matched plasma samples. Results were coherent between matrices. Thus, VAMS was shown to be a promising alternative for adherence monitoring of at least the investigated antipsychotics.

**Supplementary Information:**

The online version contains supplementary material available at 10.1007/s00216-020-03143-0.

## Introduction

A combination of antipsychotics and psychotherapy is often used to treat psychotic symptoms caused for example by schizophrenia or bipolar disorders [1, 2], but there is a wide interpatient variability concerning the response to the therapy. It is also well known that patients suffering from psychotic symptoms often show low adherence to drug therapy [3–6]. Bohlken et al. came to the conclusion after reviewing a database that only about 60% of schizophrenia and bipolar disorder patients are expected to be adherent [7]. Consequences of non-adherence are poor or nonresponse to therapy and may then result in an increase of disease burden, inpatient admission rate, suicide rate, and treatment costs [4, 8]. Currently, analytical adherence monitoring is usually performed using traditional matrices like plasma or serum obtained from venous whole blood [9–11]. However, venous whole blood sampling procedure can cause discomfort and anxiety, especially amongst the psychiatric population and can only be performed by trained personnel. Alternative sampling methods such as microsampling may overcome these disadvantages. One of the most popular methods, first described in 1963, is sampling of dried blood spots (DBS) [12]. DBS consist of small drops of capillary blood usually with unknown volume soaked on filter paper [12]. The use of DBS is promoted as easy, cost-effective, minimally invasive, and as a suitable home sampling technique. DBS were used for therapeutic drug monitoring (TDM) of antipsychotics amongst others [13, 14]. However, DBS have also some drawbacks like hematocrit (HT) effects and volume inaccuracy [15]. Therefore, further alternatives like volumetric absorptive microsampling (VAMS) have been developed. VAMS devices consist of porous hydrophilic tips attached to a plastic sample handler, which can absorb different defined volumes of finger prick blood (FPB) by wicking (see Supplementary Information (ESM) Fig. S1) [16, 17]. VAMS are expected to maintain the advantages of DBS and to overcome their limitations. VAMS has already been used for monitoring of HbA1C [18], in metabolomic studies [19], and for screening of plasma, urine, and oral fluid for drugs of abuse [20, 21]. Furthermore, VAMS was used for TDM and determination of pharmacokinetic parameters [22]. Recently, Stern et al. published a method using VAMS for the quantification of psychiatric drugs including some antipsychotics [23]. So far and to the best of our knowledge, no research article demonstrating the feasibility of VAMS for adherence monitoring was published. Therefore, the aim of this study was to develop and evaluate a VAMS-based strategy for adherence monitoring of antipsychotics in FPB by using drug concentrations to complement qualitative urine analysis [24]. Analysis should be done by means of LC-HRMS/MS and the whole quantitative workflow should be validated in accordance with international guidelines [25, 26]. Validation included the evaluation of an HT range from 20 to 60% and a quantitative comparison of VAMS to plasma samples gained from matched whole blood. The 13 most frequently prescribed antipsychotics—amisulpride, aripiprazole, cyamemazine, clozapine, haloperidol, melperone, olanzapine, paliperidone, pipamperone, promethazine, prothipendyl, quetiapine, and risperidone—should be included making the method suitable for adherence monitoring as complement to urine analysis and demonstrating its potential for TDM.

## Experimental

### Chemicals and other materials

Quetiapine was purchased from Astra Zeneca, Macclesfield (UK); promethazine from Bayer, Leverkusen (Germany); paliperidone and risperidone from Janssen, Beerse (Belgium); amisulpride, melperone, and prothipendyl from LGC, Luckenwalde (Germany); clozapine from Novartis Pharma, Wehr (Germany); haloperidol from Siegfried, Säckingen (Germany); and aripiprazole, cyamemazine, and olanzapine from Sigma-Aldrich, St. Louis (USA). Trimipramine-d_3_ was purchased from LGC, Wesel (Germany). All other chemicals (LC-MS grade or analytical grade) were from VWR, Darmstadt (Germany). Mitra VAMS with a 10-μL absorbing tip were purchased from Neoteryx, Torrance (USA). Blank EDTA blood used for development and validation of the procedure was collected from drug-free healthy volunteers after obtaining written informed consent. Blood samples for applicability studies were submitted to the authors’ laboratory for regular toxicological analysis and handled according to the institutional protocol and regulations concerning data privacy and sample handling.

### Calibrators, quality controls, internal standards, and preparation of VAMS

Stock solutions of each compound were prepared at a concentration of 1 mg/mL in methanol, except for aripiprazole, olanzapine, and prothipendyl, which were prepared in DMSO. The internal standard (IS) solution contained 0.005 mg/mL trimipramine-d_3_ in methanol. Calibrator and quality controle (QC) working solutions were prepared in methanol (stock solution A) or DMSO (stock solution B). The final concentration in whole blood used to load VAMS tips is shown in Table 1. Calibrator and QC samples were obtained by adding 10 μL of each working solution to 380 μL blank human whole EDTA blood. For the preparation of the different concentrations, the spiked solution did not exceed 5% of the total matrix volume in order not to influence the composition of the matrix [26]. The preparation of different target HT values was achieved by removing or adding plasma after centrifugation for 11 min at 9660×*g* [27, 28].

**Table 1:** Final concentrations (ng/mL) of analytes in whole blood used to load calibrator (Cal) VAMS-tips and quality control (QC) VAMS-tips as well as weighting factors used for linear regression. *LLOQ* lower limit of quantification, *ULOQ* upper limit of quantification

Analyte	Cal 1 LLOQ	Cal 2	Cal 3	Cal 4	Cal 5	Cal 6	Cal 7	Cal 8 ULOQ	QC low	QC medium	QC high	Weighting
Amisulpride	50	85	100	200	400	600	800	1000	150	500	850	1/*x*^2^
Aripiprazole	50	85	100	200	400	600	800	1000	150	500	850	1/*x*^2^
Clozapine	50	85	100	200	400	600	800	1000	150	500	850	1/*x*^2^
Cyamemazine	0.5	0.85	1	2	4	6	8	10	1.5	5	8.5	1/*x*
Haloperidol	0.5	0.85	1	2	4	6	8	10	1.5	5	8.5	Equal
Melperone	5	8.5	10	20	40	60	80	100	15	50	85	1/*x*^2^
Olanzapine	5	8.5	10	20	40	60	80	100	15	50	85	1/*x*^2^
Paliperidone	5	8.5	10	20	40	60	80	100	15	50	85	1/*x*^2^
Pipamperone	50	85	100	200	400	600	800	1000	150	500	850	Equal
Promethazine	5	8.5	10	20	40	60	80	100	15	50	85	1/*x*^2^
Prothipendyl	0.5	0.85	1	2	4	6	8	10	1.5	5	8.5	1/*x*
Quetiapine	50	85	100	200	400	600	800	1000	150	500	850	1/*x*^2^
Risperidone	0.5	0.85	1	2	4	6	8	10	1.5	5	8.5	1/*x*^2^

Whole blood was incubated for 30 min at 37 °C and 1500 rpm to allow plasma-protein binding and diffusion into red blood cells. Afterwards, VAMS tips were held onto the surface of whole blood until they turned completely red with an additional waiting time of 2 s [16]. VAMS tips were dried for 3 h at room temperature (24 °C) before sample preparation [29]. Concentrations of standard solutions used for standard addition procedure in plasma are shown in the ESM in Table S1. All solutions were stored at − 20 °C in amber glass vials.

### VAMS sample preparation

The sample preparation was based on a method published by D’Urso et al. with some adjustments [30]. Dried VAMS tips were stripped into 2-mL reaction tubes. Volumes of 90 μL purified water and 10 μL IS solution were added. Samples were shaken for 15 min at 1500 rpm and 37 °C. Afterwards, 200 μL of acetonitrile (ACN) was added and samples were shaken for 30 min at 1500 rpm and room temperature (24 °C) followed by 10 min of centrifugation at 15,000×*g* and at −10 °C. Finally, the supernatant was transferred into LC vials and a volume of 20 μL was injected onto the LC-HRMS/MS system, and analyzed as described below.

### Plasma sample preparation

Concentrations as well as addition steps used for standard addition procedure are shown in the ESM Tables S1 and S2 and extraction was based on a slightly modified procedure published by Montenarh et al. [31]. Briefly, 300 μL plasma was mixed with 300 μL of Soerensen’s buffer (pH 7.4) and 20 μL IS solution and then spiked with addition solutions. For liquid-liquid extraction, 700 μL ethylacetate:diethylether (50:50, v/v) was added and samples were shaken for 5 min, 1500 rpm at room temperature (24 °C) before centrifugation (5 min, 18,407×*g*). The supernatant was transferred to a vial and evaporated under a gentle flow of nitrogen at 40 °C. The remaining aqueous layer was mixed with 150 μL NaOH (1 M) and 700 μL ethylacetate:diethylether (50:50, v/v) and shaken for 5 min at 1500 rpm at room temperature (24 °C) before centrifugation (5 min, 18,407×*g*). The supernatant was transferred to the previously used vial and also evaporated under a gentle flow of nitrogen at 40 °C. The extracts were finally reconstituted in 100 μL of methanol and 20 μL was injected onto the LC-HRMS/MS system as described below.

### Instrumental settings

All samples were analyzed using a Thermo Fisher Scientific (TF, Dreieich, Germany) Dionex UltiMate 3000 consisting of a degasser, a quaternary pump, a DL W2 wash system, and an HTC PAL autosampler (CTC Analytics AG, Zwinger, Switzerland), coupled to a TF Q-Exactive system equipped with a heated electrospray ionization (HESI)-II source. Mass calibration was done prior to analysis according to the manufacturer’s recommendations using external mass calibration. Gradient elution was performed on a TF Accucore phenyl-hexyl column (100 mm × 2.1 mm, 2.6-μm particle size). The mobile phase A consisted of 2 mM aqueous ammonium formate containing formic acid (0.1%, v/v, pH 3), and mobile phase B consisted of 2 mM aqueous ammonium formate with acetonitrile:methanol (50:50, v/v) plus formic acid (0.1%, v/v), and water (1%, v/v). The flow rate was set at 750 μL/min. The gradient was programmed as follows: 0–1 min from 100% A to 95% A, 1–4.2 min to 90% A, 4.2–13.5 min to 85% A, 13.5 to 18 min to 65% A, 18–19 min to 1% A, 19–21.8 min hold 1% A, 21.8 min to 25 min hold 100% A. Chromatography was performed at 70 °C. The HESI-II source conditions were as follows: ionization mode, positive; sheath gas flow rate, 60 arbitrary units (AU); auxiliary gas flow rate, 10 AU; spray voltage, 4.00 kV; auxiliary gas heater temperature, 360 °C; ion transfer capillary temperature, 320 °C; and S-lens RF level, 60.0.

Mass spectrometry was performed using parallel reaction monitoring (PRM) with an inclusion list containing masses of interest in defined time windows. The settings for PRM data acquisition were as follows: resolution, 17,500; microscans, 1; automatic gain control (AGC) target, 2e5; maximum injection time (IT), 250 ms; scan range, *m*/*z* 170 to 930; isolation window, 2.0 *m*/*z*; HCD with stepped normalized collision energy (NCE), 17.5, 35, and 52.5%; spectrum data type, profile. TF Xcalibur Qual Browser software version 2.2 was used for data handling. Precursor ion masses (*m*/*z*) used for the inclusion list are represented in the ESM Table S3.

### Method validation

The validation was performed according to published recommendations including the European Medicines Agency (EMA) Guideline on Bioanalytical Method Validation and the International Association of Therapeutic Drug Monitoring and Clinical Toxicology (IATDMCT) guideline for development and validation of dried blood spot–based methods for therapeutic drug monitoring [25, 26]. The statistical evaluation of the peak area ratios of analytes versus IS was performed using Microsoft Excel version 16 (Microsoft, Redmond, USA) and TF Xcalibur Quan Browser version 2.2.

For selectivity testing, 13 blank matrices from individual donors were analyzed in PRM mode for possible peak interferences of analytes or IS. These were divided in 10 drug-free FPB donors and three samples were loaded with EDTA blood–containing drugs but no antipsychotics. For carry-over testing, a blank matrix sample was injected after analysis of the highest calibrator (*n* = 3). Interfering signals of analytes in blank matrix should be less than 20% of the lower limit of quantification (LLOQ) and less than 5% of the IS for selectivity and carry-over testing.

Three calibration ranges were defined consisting of eight calibration points, each. Calibrators were prepared by spiking blank matrix with different calibrator solutions (final matrix concentrations; see Table 1). Extraction was performed as described before. All LLOQ and upper limits of quantification (ULOQ) are shown in Table 1. Different weighting factors (equal, 1/*x*, or 1/*x*^2^) for linear regression were tested by fitting three curves of each analyte. The suitability of the models was evaluated using the square sum of residuals (SSR) by choosing the model with the lowest SSR. All calculations were performed using KNIME (www.knime.org) and R snippets (www.r-projects.org). Back-calculated concentrations of calibrators should be within ± 15% of the nominal value (LLOQ ± 20%). If criteria are not met, the calibration standard should be rejected for regression analysis. At least 75% of the calibration standards must fulfill criteria.

Accuracy and precision of the quantification results were determined for the LLOQ, QC low, QC medium, and QC high levels listed in Table 2. Therefore, QC samples were spiked with separately prepared stock solutions from calibration standards. QC sample concentrations were back-calculated via the calibration curves and compared with their nominal values. The evaluation was performed for values of five sample replicates obtained within a single run (within-run accuracy and precision) and in three different runs (between-run accuracy and precision). For positive assessment of accuracy, mean concentrations should be within ± 15% of the nominal values (± 20% for LLOQ). For a positive assessment of precision, the coefficient of variation (CV) should be within 15% (20% for LLOQ).

**Table 2:** Within- and between-day accuracy (A) and precision (P) of the LLOQ, QC low, QC medium, and QC high. Dilution integrity with acetonitrile containing IS (ACN) or with processed blank matrix containing IS (matrix). 1:5 dilution of a 2.5 higher concentration than the highest calibrator. *LLOQ* lower limit of quantification, *QC* quality control, *IS* internal standard; *mid* medium

Analyte	Relative mean concentration (A), % (CV (P), %)									
	Within-day				Between-day				Dil 1:5	
	LLOQ	QC low	QC mid	QC high	LLOQ	QC low	QC mid	QC high	ACN	Matrix
Amisulpride	106 (8)	102 (8)	93 (6)	107 (7)	105 (8)	102 (11)	95 (6)	104 (11)	69 (9)	99 (5)
Aripiprazole	100 (2)	100 (9)	109 (5)	97 (4)	99 (10)	106 (8)	105 (5)	100 (8)	67 (7)	105 (7)
Clozapine	97 (8)	107 (8)	102 (6)	115 (6)	100 (6)	106 (7)	103 (5)	111 (8)	77 (7)	115 (4)
Cyamemazine	90 (13)	95 (9)	88 (7)	90 (7)	115 (27)	87 (17)	84 (7)	90 (9)	56 (12)	90 (7)
Haloperidol	105 (15)	95 (9)	100 (5)	103 (5)	106 (17)	101 (12)	98 (7)	104 (11)	80 (12)	106 (9)
Melperone	107 (4)	101 (8)	96 (7)	98 (7)	97 (10)	96 (9)	95 (6)	101 (10)	61 (9)	89 (7)
Olanzapine	89 (14)	101 (11)	98 (6)	100 (2)	92 (11)	98 (11)	99 (6)	101 (10)	38 (13)	50 (12)
Paliperidone	96 (8)	110 (13)	102 (8)	109 (5)	102 (9)	109 (11)	104 (6)	109 (10)	71 (8)	107 (4)
Pipamperone	96 (7)	113 (11)	100 (6)	111 (7)	99 (7)	111 (9)	106 (7)	110 (9)	65 (8)	110 (3)
Promethazine	97 (8)	104 (4)	97 (6)	104 (9)	101 (11)	102 (7)	96 (6)	105 (10)	65 (11)	97 (8)
Prothipendyl	120 (10)	97 (12)	102 (9)	100 (9)	111 (38)	98 (12)	106 (9)	103 (10)	70 (13)	104 (8)
Quetiapine	101 (3)	105 (10)	96 (4)	105 (3)	108 (14)	106 (10)	100 (6)	106 (12)	72 (6)	110 (4)
Risperidone	98 (15)	95 (8)	86 (2)	91 (4)	103 (12)	97 (11)	89 (5)	96 (11)	64 (7)	94 (4)

Dilution integrity of the final extract was determined by spiking blank samples (*n* = 5) at 2.5 times higher than the highest calibrator. Extracts were diluted 1:5 with IS-spiked ACN or processed blank matrix containing IS [26]. Since prothipendyl shows a broad therapeutic range (ESM Table S4), two additional dilution factors were tested for this analyte. An extracted sample stemming from a blood concentration of 100 ng/mL prothipendyl was diluted 1:10 and 1:20 with either IS-spiked ACN or processed blank matrix containing IS. All samples were analyzed, and accuracy and precision were determined.

The matrix factor (MF) and the recovery (RE) were investigated at low and high QC levels for six blank matrix samples of individual donors. VAMS tips were prepared with whole blood (EDTA) of HT 40%. Additionally, three blank matrix samples of individual donors were prepared at HT 20% and HT 60% each. The MF was calculated by the ratio of the peak area in the presence of matrix (blank matrix spiked after extraction) to the peak area in the absence of matrix (pure analyte solution). The IS-normalized MF was calculated by dividing the MF of the analytes by the MF of the IS. The RE was calculated by the ratio of the peak area extracted with the matrix (blank matrix spiked before extraction) to the peak area in the presence of matrix (blank matrix spiked after extraction). The CV of the IS-normalized MF and the RE should be within 15%.

The stability of the stock solutions A and B was tested over a time of 10 weeks (*n* = 3). Furthermore, autosampler stability (24 h, 10 °C) of processed samples and long-term stability (1 and 2 weeks, 24 °C) in the test device were investigated (*n* = 4) using the low and high QC levels. Deviations between obtained and nominal concentrations of QCs should be within ± 15% when analyzed immediately after preparation and after evaluated storage conditions using a freshly prepared calibration curve.

### Proof of concept

Matched human plasma samples and VAMS tips soaked with EDTA blood of 10 individuals were analyzed. All samples were submitted to the authors’ laboratory for regular toxicological analysis.

## Results and discussion

Assessing adherence based on serum drug concentrations to complement urine analysis is expected to be more accurate than urine analysis alone, particularly when using the so-called dose-related concentration approach as proposed, e.g., by Ritscher et al. [24]. In general, drugs with low bioavailability, low renal excretion, or underlying extensive metabolism may be underestimated concerning their adherence when using qualitative urine analysis alone. Therefore, we aimed to develop a strategy for the simultaneous quantification of the 13 most frequently prescribed antipsychotics in FPB using VAMS with a particular focus on adherence monitoring. Paliperidone (9-hydroxyrisperidone) can be prescribed as a drug but is also the active metabolite of risperidone. Aripiprazole, clozapine, and quetiapine also have active metabolites; however, none of them can be prescribed as a drug itself. For a correct assessment of adherence, the quantification of active metabolites is not necessary. However, this might be a limitation point if the method is used for TDM.

Figures S2 and S3 (ESM) show the chromatographic separation of the antipsychotics at the LLOQ level and in a patient sample within 20 min, respectively. The long run time of 20 min (including the equilibration step of then in total 25 min) was acceptable as the method could be integrated into our laboratory routine. The aim was to achieve sufficient separation without changing the column type or composition of mobile phases and to retain the standard setup. Furthermore, adherence monitoring is normally no urgent analysis, and therefore a total runtime of 25 min was acceptable. An injection volume of 20 μL was chosen since it provided better sensitivity compared to a volume of 10 μL. This in turn led to peak-decompression, which was tolerated and had no negative impact on analytical results. For identification, full MS^2^-spectra were compared to a database [32] and the peak area ratios of the analyte-specific fragment ion in MS^2^ (ESM Table S3) and those of the IS were used for quantification. For analysis of human samples, each analytical batch consisted of a zero-sample containing IS but no antipsychotics, eight calibration standards, three QC levels, and the patient samples.

The IS trimipramine-d_3_ did not coelute with the analytes. No interfering signals from endogenous compounds or other drugs (listed in ESM Table S5) as well as no IS carry-over could be observed. However, carry-over was occasionally observed for cyamemazine, haloperidol, and olanzapine especially after injection of concentrations above the therapeutic range. Hence, samples following a high concentration of those drugs should be reanalyzed after a washing run with extracted blank matrix.

A linear calibration model could be used for all analytes after testing different weighting factors (equal, 1/*x*, 1/*x*^2^; see Table 1). Results for the within- and between-day accuracy and precision are shown in Table 2. For the within-day accuracy, all analytes had a mean concentration within ± 15% (LLOQ ± 20%) of the nominal concentration and the within-day precision did not exceed a CV of 15%. However, for between-day accuracy and precision, cyamemazine only met required validation parameters at the high QC level and prothipendyl exceeded precision at the LLOQ. Anyhow, the lowest therapeutic concentration of prothipendyl is already covered by the QC low level. Consequently, the QC low level should be used as cutoff concentration for the quantification of prothipendyl. Cyamemazine failed the validation in this assay.

Dilution integrity was tested by dilution (1:5) of a processed sample, having a concentration 2.5 times higher than the highest calibrator, either with IS-spiked ACN (trimipramine-d_3_, 0.166 mg/L) or with processed blank matrix containing IS. Using IS-spiked ACN for dilution, none of the analytes passed validation. However, if the final extract was diluted with processed blank matrix containing IS, all analytes passed validation except olanzapine. Anyway, the therapeutic range of olanzapine is completely covered by the calibration range. For prothipendyl, two additional dilution factors (1:10 and 1:20) were tested. Again, validation parameters were only fulfilled when processed blank matrix containing IS was used (see ESM Table S6).

Determined IS-normalized MFs and CVs are given in Table 3. At different HT values, MFs varied between 0.77 and 1.28. However, these effects were found to be reproducible with CVs within 15% except for haloperidol (QC low HT 20% and HT 60%), olanzapine (QC low and high HT 60%), and prothipendyl (QC low HT 20% and HT 60%). Thus, the MF was reproducible for all analytes at HT 40%. Higher CVs at HT 20% and HT 60% for haloperidol, olanzapine, and prothipendyl should not play a role for normal HT fluctuation of healthy patients.

**Table 3:** Internal standard (IS)–normalized matrix factors (MF), recovery (RE), and coefficients of variation (CVs) of the analytes of low- and high-quality controls (QC) for VAMS at different hematocrit (HT) values (*n* = 6 at HT 40%; n=3 at HT 20% and HT 60%)

Analyte	IS-normalized MF (CV, %)						RE, % (CV, %)					
	QC low			QC high			QC low			QC high		
	HT 20%	HT 40%	HT 60%	HT 20%	HT 40%	HT 60%	HT 20%	HT 40%	HT 60%	HT 20%	HT 40%	HT 60%
Amisulpride	0.85 (7)	0.84 (6)	0.98 (7)	0.83 (7)	0.86 (7)	0.87 (10)	138 (8)	123 (6)	132 (13)	146 (7)	130 (4)	132 (3)
Aripiprazole	0.97 (5)	1.02 (8)	1.15 (2)	1.05 (4)	1.05 (8)	1.08 (10)	131 (8)	123 (8)	117 (4)	128 (4)	118 (5)	110 (6)
Clozapine	0.95 (6)	0.92 (6)	1.06 (4)	0.91 (5)	0.91 (6)	0.95 (9)	111 (5)	114 (9)	109 (3)	117 (4)	105 (7)	102 (6)
Cyamemazine	1.24 (9)	1.28 (12)	1.26 (1)	1.14 (8)	1.09 (8)	1.10 (6)	49 (13)	52 (10)	63 (18)	68 (9)	68 (8)	58 (4)
Haloperidol	0.94 (20)	0.99 (14)	1.08 (33)	0.93 (8)	0.95 (3)	0.92 (5)	71 (2)	93 (12)	103 (14)	80 (12)	75 (13)	91 (7)
Melperone	0.85 (4)	0.85 (5)	0.95 (6)	0.89 (7)	0.89 (5)	0.94 (8)	63 (15)	67 (11)	65 (15)	54 (10)	60 (11)	61 (6)
Olanzapine	0.77 (10)	0.81 (10)	0.93 (20)	0.86 (7)	0.85 (7)	0.94 (17)	26 (5)	13 (18)	16 (21)	34 (15)	26 (18)	20 (14)
Paliperidone	0.88 (6)	0.87 (7)	1.04 (12)	0.90 (4)	0.91 (5)	0.95 (11)	112 (3)	132 (9)	138 (12)	136 (3)	126 (8)	123 (8)
Pipamperone	0.99 (4)	1.00 (5)	1.12 (4)	0.99 (7)	0.98 (6)	1.01 (9)	114 (8)	114 (7)	123 (8)	115 (3)	115 (4)	112 (5)
Promethazine	0.92 (5)	0.92 (6)	1.01 (8)	0.97 (4)	0.96 (5)	1.02 (9)	60 (12)	57 (14)	56 (11)	59 (6)	55 (7)	52 (7)
Prothipendyl	0.96 (18)	1.10 (14)	1.06 (22)	0.96 (4)	0.91 (11)	0.99 (6)	32 (20)	62 (16)	63 (4)	45 (9)	46 (15)	39 (6)
Quetiapine	0.88 (1)	0.90 (5)	1.00 (3)	0.91 (8)	0.91 (8)	0.93 (7)	146 (7)	139 (8)	137 (3)	143 (4)	133 (4)	129 (4)
Risperidone	0.85 (3)	0.85 (5)	0.91 (7)	0.85 (7)	0.89 (6)	0.91 (9)	116 (14)	107 (6)	125 (3)	123 (5)	117 (11)	114 (5)

RE values of the analytes of low and high QC in VAMS tips at different HT values are summarized in Table 3. The RE was found to be reproducible, even at low HT values, with CVs within 15% except for cyamemazine (QC low HT 60%), olanzapine (QC low HT 40% and HT 60%; QC high HT 40%), and prothipendyl (QC low HT 20%). However, CVs never exceeded 21%. Only for a few antipsychotics, the nominal RE value at HT 20% and 60% differed from the HT 40% value by more than 15%. Greater nominal RE deviations at the low HT values were observed for amisulpride (QC high HT 20%), haloperidol (QC low HT 20% and QC high HT 60%), paliperidone (QC low HT 20%), prothipendyl (QC low HT 20%), and risperidone (QC low HT 60%). Only in the case of haloperidol (21%) and prothipendyl (30%), nominal RE deviation exceeded 20%. In summary, the present method allowed the quantification of amisulpride, aripiprazole, clozapine, haloperidol, melperone, olanzapine, paliperidone, pipamperone, promethazine, prothipendyl, quetiapine, and risperidone over their complete therapeutic range (ESM Table S4) in whole blood (HT 40%) using VAMS devices. For prothipendyl, concentration exceeding the QC high can be quantified since dilution integrity was given for three dilution factors. Only cyamemazine failed validation in between-day accuracy and precision.

Stock solutions were stable over 10 weeks at −20 °C in amber glass vials. Results of short- and long-term stability are given in ESM Table S7. After storage of processed samples in the autosampler for 24 h at 10 °C, no analyte showed a degradation over 15% of the nominal concentration for the low and high QC levels. This is of advantage if long-lasting analytical series are planned. However, autosampler stability can only be guaranteed for 24 h, based on these experiments. Hence, the duration of a batch should not exceed this period of time. However, not all analytes are long-term stable in the device. After 1-week storage at 24 °C, cyamemazine, melperone, olanzapine, promethazine, and prothipendyl showed degradation over 15% of nominal concentration. Haloperidol was stable for 1 week and amisulpride, aripiprazole, clozapine, paliperidone, pipamperone, quetiapine, and risperidone were stable for 2 weeks in the sample device at 24 °C. The instability of olanzapine and promethazine in whole blood at 20 °C was already described but melperone was stable over 10 weeks at 20 °C in whole blood [33]. Good stability of amisulpride, aripiprazole, clozapine, haloperidol, paliperidone, pipamperone, quetiapine, and risperidone was already described for DBS [14, 34, 35]. Proof of concept of the presented method was achieved using 10 matched human whole blood (EDTA)–loaded VAMS tips and plasma samples. Unfortunately, samples containing aripiprazole, cyamemazine, haloperidol, and prothipendyl were not available for further evaluation.

Table 4 shows the results after analyzing VAMS tips and plasma and the assessment of adherence in both matrices. For a positive assessment of adherence, the minimal concentration in blood should be above the lowest published therapeutic concentration with a tolerance limit of ± 15% to take measurement inaccuracy into account. Adherence assessment differed between VAMS and plasma only once. This could be explained by the measurement inaccuracy within ± 15% regarding plasma. In three cases, quantification revealed concentrations above the therapeutic range but it is known that high doses of antipsychotics are used for treatment [36, 37]. Results also showed that VAMS can be a promising tool for adherence monitoring and that the developed VAMS strategy is consistent with findings in plasma.

**Table 4:** Quantification of antipsychotics (ng/mL) using VAMS tips and plasma as sample matrix and assessment of adherence. *conc.* concentration

Analyte	Conc. in VAMS (ng/mL)	Adherence assessment in VAMS	Conc. in plasma (ng/mL)	Adherence assessment in plasma
Amisulpride	209	Adherent	216	Adherent
Clozapine	400	Adherent	368	Adherent
Olanzapine	93*	Adherent	98*	Adherent
Olanzapine	64	Adherent	72	Adherent
Paliperidone	< 5	Non-adherent	6	Non-adherent
Paliperidone	12	Non-adherent	12	Non-adherent
Pipamperone	75	Non-adherent	115	Adherent
Pipamperone	450*	Adherent	505*	Adherent
Pipamperone	< 50	Non-adherent	58	Non-adherent
Pipamperone	< 50	Non-adherent	15	Non-adherent
Promethazine	18	Adherent	10	Adherent
Promethazine	36	Adherent	39	Adherent
Quetiapine	< 50	Non-adherent	8	Non-adherent
Quetiapine	< 50	Non-adherent	94	Non-adherent
Quetiapine	952*	Adherent	1082*	Adherent
Risperidone	5	Adherent	10	Adherent
Risperidone	5	Adherent	5	Adherent

*Above the therapeutic range

Since the method was fully validated and meets certain requirements such as a full calibration within therapeutic ranges of the antipsychotics, it might also be used for TDM. However, before using this method for TDM purpose, some more data are needed. The therapeutic ranges given in ESM Table S4 are plasma concentrations and the relationship between plasma and whole blood concentrations has to be established first. Patteet et al. calculated whole blood therapeutic ranges using blood to serum ratios and conducted a clinical study to make a comparison between antipsychotic concentrations found in venous whole blood and capillary DBS samples. They came to the result that the clinical interpretation, for the tested antipsychotics, of serum, whole blood, and DBS concentrations was identical [38]. Stern et al. also compared capillary blood, whole blood, and plasma concentrations of psychiatric drugs and came to the result that the three matrices showed a good correlation between determined concentrations [23]. However, exact concentrations including converting factors are not required for the assessment of adherence, which was the aim of the current study.

## Conclusions

A VAMS- and LC-HRMS/MS-based workflow for simultaneous quantification of 13 antipsychotics was successfully developed and validated. Only 10 μL of blood was required and the straightforward sample preparation was followed by a complete chromatographic separation. Proof of concept was demonstrated by quantification of 17 intakes of antipsychotics in VAMS tips and matched plasma samples of patients. VAMS as a sampling strategy for dried matrix was able to overcome the hematocrit issue in most cases but the advantage of stability in dried matrix could only be proven for half of the analytes.

## Supplementary information

ESM 1 (PDF 326 kb)
