# Supplementary material for: Development, validation, and application of a quantitative volumetric absorptive microsampling–based method in finger prick blood by means of LC-HRMS/MS applicable for adherence monitoring of antipsychotics
Source: Anal Bioanal Chem. 2021 Jan 30;413(6):1729–37. doi: 10.1007/s00216-020-03143-0 (PMC7921024; doi:10.1007/s00216-020-03143-0)
Supplement: Supplementary file 1 — (PDF 326 kb) [file 216_2020_3143_MOESM1_ESM.pdf]

Electronic supplementary material

Development, validation, and application of a quantitative volumetric absorptive microsampling-based method in finger prick blood by means of LC-HRMS/MS applicable for adherence monitoring of antipsychotics

Cathy M. Jacobs, Lea Wagmann, Markus R. Meyer*

Department of Experimental and Clinical Toxicology, Institute of Experimental and Clinical Pharmacology and Toxicology, Center for Molecular Signaling (PZMS), Saarland University, Homburg, Germany

Table S1: Concentrations in mg/L of standard solutions used for standard addition procedure in plasma

**Analyte**	**Standard solution A mg/L in methanol**	**Standard solution B mg/L in DMSO**
Amisulpride	6	-
Aripiprazole	-	6
Clozapine	6	-
Cyamemazine	0.06	-
Haloperidol	0.06	-
Melperone	0.6	-
Olanzapine	-	0.6
Paliperidone	0.6	-
Pipamperone	6	-
Promethazine	0.6	-
Prothipendyl	-	0.06
Quetiapine	6	-
Risperidone	0.06	-

Table S2: Sample preparation for standard addition procedure (MeOH: methanol)

**Addition step**	**Sample volume, µL**	**Standard solution A, µL**	**Standard solution B, µL**	**MeOH, µL**
0	300	0	0	114
1	300	10	10	94
2	300	34	34	46
3	300	57	57	0

Table S3: Precursor ion masses used in the inclusion list for positive ionization mode, fragment ion mass used for quantification, and retention times of the neuroleptics, and internal standard (IS)

**Analyte**	**Precursor ion mass, *m/z***	**Fragment ion mass, *m/z* for quantification**	**Retention time, min**
Amisulpride	370.1795	242.0479	4.2
Aripiprazole	448.1553	285.0916	19.1
Clozapine	327.1371	270.0791	10.7
Cyamemazine	324.1529	100.1124	17.5
Haloperidol	376.1474	165.0711	16.5
Melperone	264.1758	165.0710	7.4
Olanzapine	313.1481	256.0910	2.9
Paliperidone	427.2140	207.1124	8.8
Pipamperone	376.2395	165.0712	5.3
Promethazine	285.1420	86.0970	15.9
Prothipendyl	286.1372	241.0796	13.1
Quetiapine	384.1740	253.0789	14.8
Risperidone	411.2191	191.1179	11.5
Trimipramine-d_3 (IS)	298.2357	103.1312	18.4

Table S4: Therapeutic plasma concentrations (ng/mL) of neuroleptics. C_{min} -15%: minimal therapeutic concentration with a subtraction of 15%.

**Analyte**	**Therapeutic range, ng/mL**			**C_{min} -15%, ng/mL**
	**Schulz et al. 2012 [1]**	**Schulz et al. 2020 [2]**	**AGNP, 2017 [3]**	
Amisulpride	100-400	100-400	100-320	85
Aripiprazole	150-500	100-350	100-350	85
Clozapine	350-600	350-600	350-600	297
Cyamemazine	-	0.9-1.6	-	0.77
Haloperidol	5-17	5-17	1-10	0.85
Melperone	30-100	30-100	30-100	25.5
Olanzapine	20-80	20-80	20-80	17
Paliperidone	20-60	20-60	20-60	17
Pipamperone	100-400	100-400	100-400	85
Promethazine	50-200	10-50	-	8.5
Prothipendyl	5-10	30-80	30-80	4.25
Quetiapine	100-500	100-500	100-500	85
Risperidone	2-20	2-20	-	1.70

Table S5: Drugs included in the selectivity studies

Amlodipine
Brotizolam
Cafedrine
Doxazosin
Finasteride
Metamizole
Midazolam
Nortilidine
Pantoprazole
Tamsulosin
Torasemide

Table S6: Dilution integrity for a sample containing prothipendyl at a concentration of 100 ng/mL. Dilution was performed with acetonitrile containing IS (ACN) or with processed blank matrix containing IS (matrix)

**Dilution factor**	**Relative mean concentration, % (CV, %)**	
	**ACN**	**Matrix**
1:10	71 (8)	100 (8)
1:20	78 (7)	102 (7)

Table S7: Autosampler stability at 10°C and one and two-week stability in the sampling device at 24°C (n=4)

**Analyte**	**Relative mean concentration, %**					
	**QC low**			**QC high**		
	**24h 10°C**	**1 week 24°C**	**2 weeks 24°C**	**24h 10°C**	**1 week 24°C**	**2 weeks 24°C**
Amisulpride	85	88	85	110	97	89
Aripiprazole	113	89	90	101	93	86
Clozapine	100	90	86	107	87	87
Cyamemazine	104	65	21	103	61	46
Haloperidol	85	98	70	92	87	76
Melperone	94	54	46	92	49	42
Olanzapine	99	39	40	99	36	27
Paliperidone	93	92	89	106	98	91
Pipamperone	98	92	98	122	99	90
Promethazine	107	45	31	103	39	29
Prothipendyl	90	10	26	101	43	40
Quetiapine	115	112	105	105	95	87
Risperidone	100	96	98	101	85	89

Figure S1: Volumetric absorptive microsampling (VAMS) device

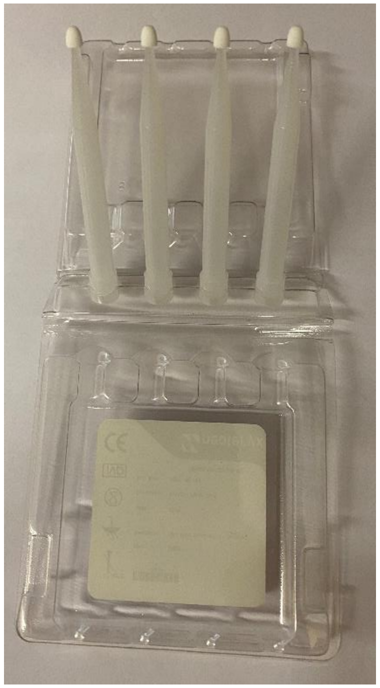

Figure S2: Chromatographic separation of all analytes at the lower limit of quantification. a: all Peaks at 100% relative abundance; b: zoom in of peaks at fixed scale

a

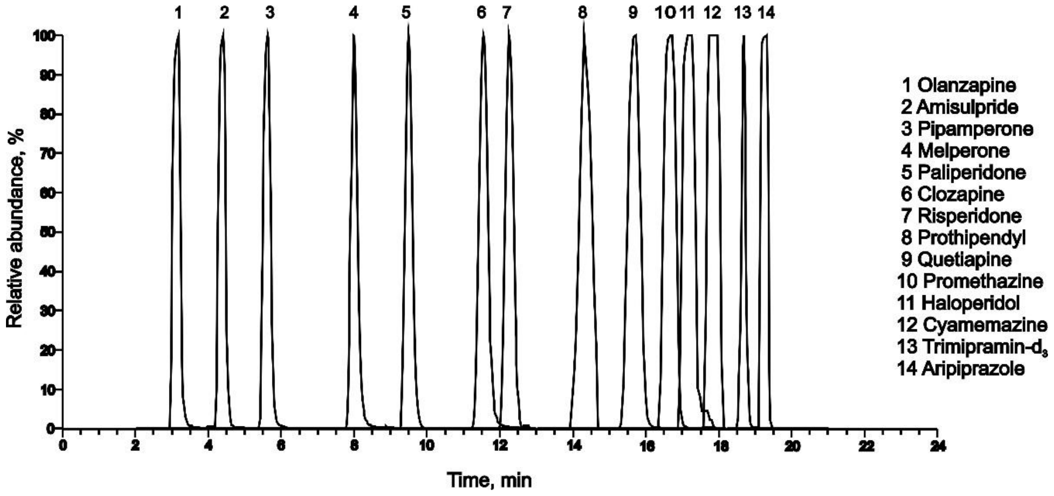

b

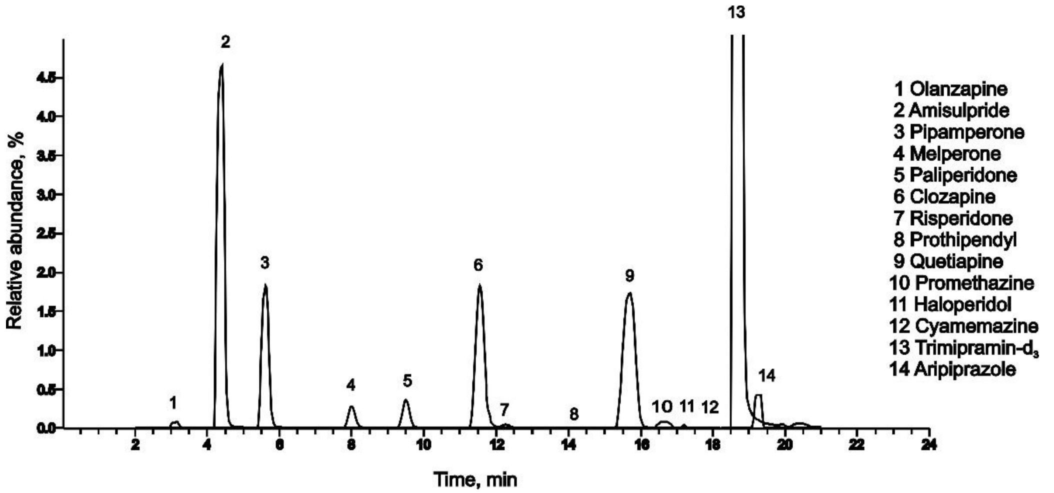

Figure S3: Chromatographic separation of analytes present in a patient sample submitted to the authors´ laboratory for adherence monitoring. All peaks are set to 100% relative abundance.

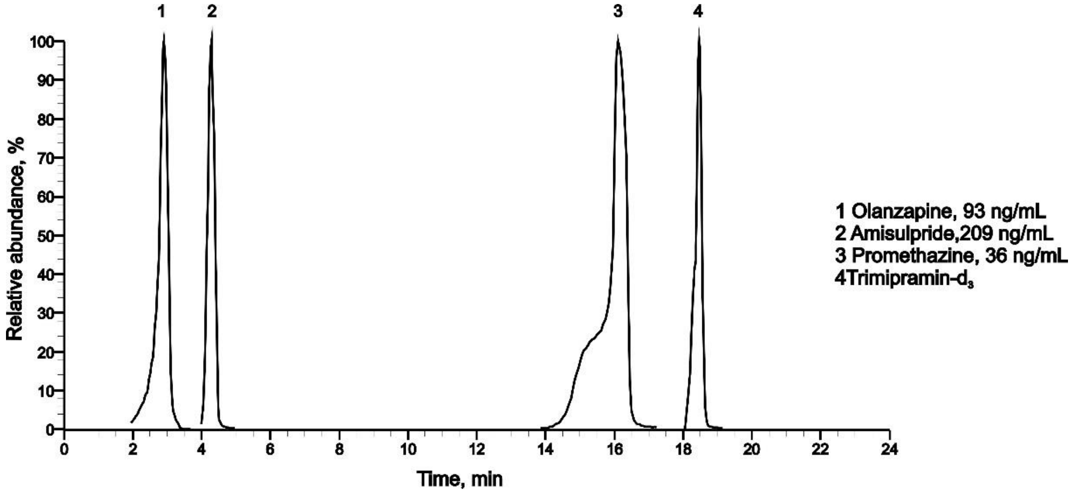

References

1. Schulz M, Iwersen-Bergmann S, Andresen H, Schmoldt A. Therapeutic and toxic blood concentrations of nearly 1,000 drugs and other xenobiotics. Crit Care. 2012;16(4):R136. doi:10.1186/cc11441.
2. Schulz M, Schmoldt A, Andresen-Streichert H, Iwersen-Bergmann S. Revisited: Therapeutic and toxic blood concentrations of more than 1100 drugs and other xenobiotics. Crit Care. 2020;24(1):195. doi:10.1186/s13054-020-02915-5.
3. Hiemke C, Bergemann N, Clement HW, Conca A, Deckert J, Domschke K et al. Consensus Guidelines for Therapeutic Drug Monitoring in Neuropsychopharmacology: Update 2017. Pharmacopsychiatry. 2017. doi:10.1055/s-0043-116492.
